# Acute fissuration of a giant splenic artery aneurysm detected by point-of-care ultrasound: case report

**DOI:** 10.1186/s13089-018-0086-3

**Published:** 2018-02-01

**Authors:** Philippe Le Conte, David Trewick, Philippe Pes, Eric Frampas, Eric Batard, Winfocus-France Group

**Affiliations:** 1 Service des Urgences, CHU de Nantes, 44093 Nantes, France; 2 Radiologie centrale, CHU de Nantes, Nantes, France

## Abstract

**Background:**

Epigastric pain is frequent in Emergency Medicine and remains a challenging situation. Besides benign etiologies such as gastritis or uncomplicated cholelithiasis, it could reveal myocardial infarction or vascular disease. Point-of-care ultrasound (POCUS) could be performed in such situation.

**Case presentation:**

A healthy 66-year-old man with no previous medical history was admitted to the Emergency Department for a rapid onset epigastric pain. He reported taking non-steroidal anti-inflammatories for 1 week prior to admission. His pain had rapidly subsided and the physical examination was inconclusive. ECG and blood samples were normal. POCUS revealed a vascular mass located between the spleen and the left kidney measuring 80 * 74 mm associated with small amounts of free peritoneal fluid. Computed tomography diagnosed a fissurated giant aneurysm of the splenic artery. The aneurysm was managed emergently by endovascular exclusion by selective splenic artery embolization. The post-intervention course was uneventful and the patient was discharged home 3 days later. The patient has remained free from any complications of the embolization 6 months after the procedure.

**Conclusion:**

Spontaneously regressive epigastric pain with a normal physical and biology/ECG should not necessarily reassure the physician, in particular if patients have cardiovascular risk factors. A POCUS should be considered for these patients.

**Electronic supplementary material:**

The online version of this article (10.1186/s13089-018-0086-3) contains supplementary material, which is available to authorized users.

## Background

Epigastric pain is a frequent and often challenging situation in the Emergency Department (ED) [1]. Besides benign etiologies such as gastritis or uncomplicated cholelithiasis, it could reveal myocardial infarction, aortic, or vascular diseases. The risk is neglecting vascular dissection or rupture leading to a possible fatal hemorrhagic shock [2]. Splenic artery aneurysms (SAA) are rare, often asymptomatic, and usually discovered incidentally [3] but remain potentially life threatening lesions [4]. SAA were reported in 0.78% of 3600 non-selective angiograms [4]; the incidence increases with female gender, in older patients and portal hypertension [5]. The ED incidence was reported as 0.011% in a retrospective study [6].

We report a case of a patient consulting in the ED for epigastric pain in whom point-of-care ultrasound (POCUS) revealed an acute fissuration of a giant splenic aneurysm.

## Case report

**Fig. 1:**
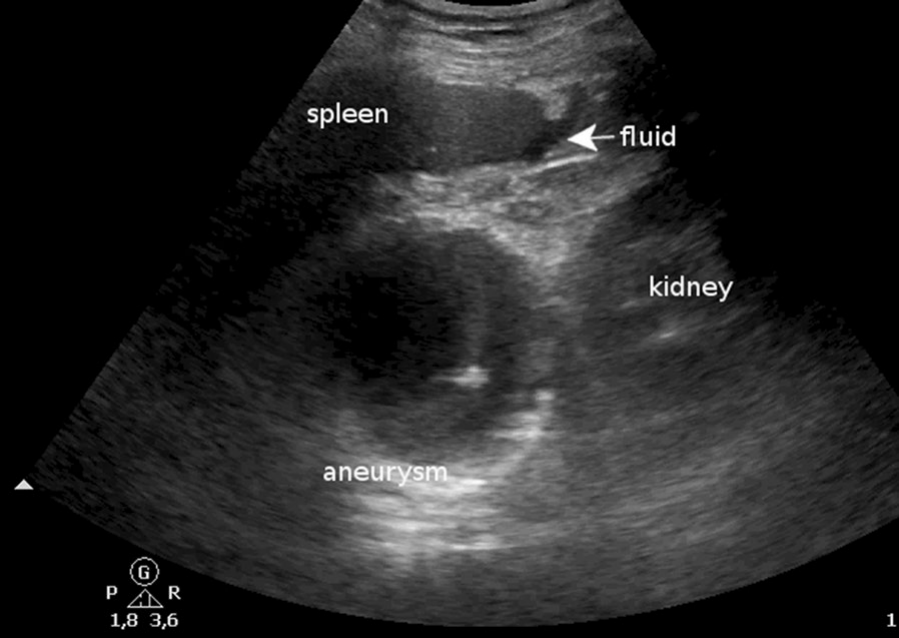
POCUS scan of the left upper quadrant of a patient presenting with epigastric pain. See the vascular mass located between spleen and kidney

**Fig. 2:**
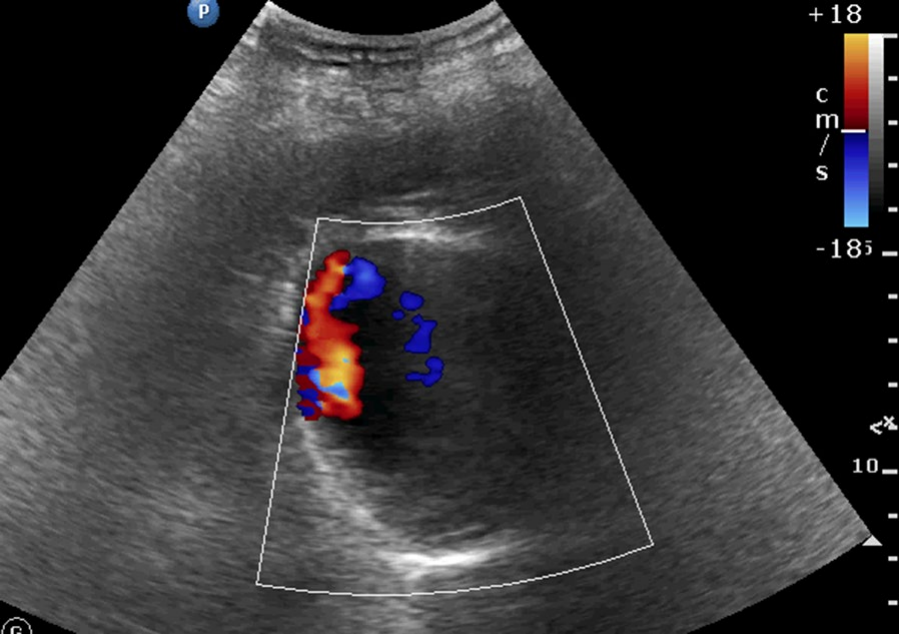
POCUS color Doppler scan of left upper quadrant in a patient presenting an epigastric pain. See the strong Doppler signal

A healthy 66-year-old man with no previous medical history was admitted to the ED for a first episode of rapid onset severe epigastric pain. It appeared at rest without associated symptoms such as dyspnea or vomiting. He reported taking non-steroidal anti-inflammatories for 1 week prior to admission for an epicondylitis. On admission, his pain had rapidly subsided and the physical examination was inconclusive: blood pressure, temperature, heart rate, and capillary filling were normal. Thoracic auscultation and abdominal palpation revealed only slight epigastric pain. ECG and troponin assay were normal as was hemoglobin, bilirubin, liver enzymes, and lipase levels. As a part of our department’s policy, a POCUS was performed essentially to rule out cholelithiasis. It was performed using a Philips CX50 with a 3.5–5 MHz abdominal probe. There were no gallstones; however, POCUS revealed a mass located between the spleen and the left kidney (Fig. 1 and Additional file 1: Video 1) measuring 80 * 74 mm. The mass was vascular with a strong color Doppler signal (Fig. 2 and Additional file 2: Video 2) and an intraluminal thrombus which explains why the Doppler signal does not fill all the lumen. The maximum velocity in the aneurysm was only 18 cm/s because of the large diameter. The aorta was explored from diaphragm to bifurcation. No other aneurysm was found. Small amounts of free peritoneal fluid were detected around the spleen.

**Fig. 3:**
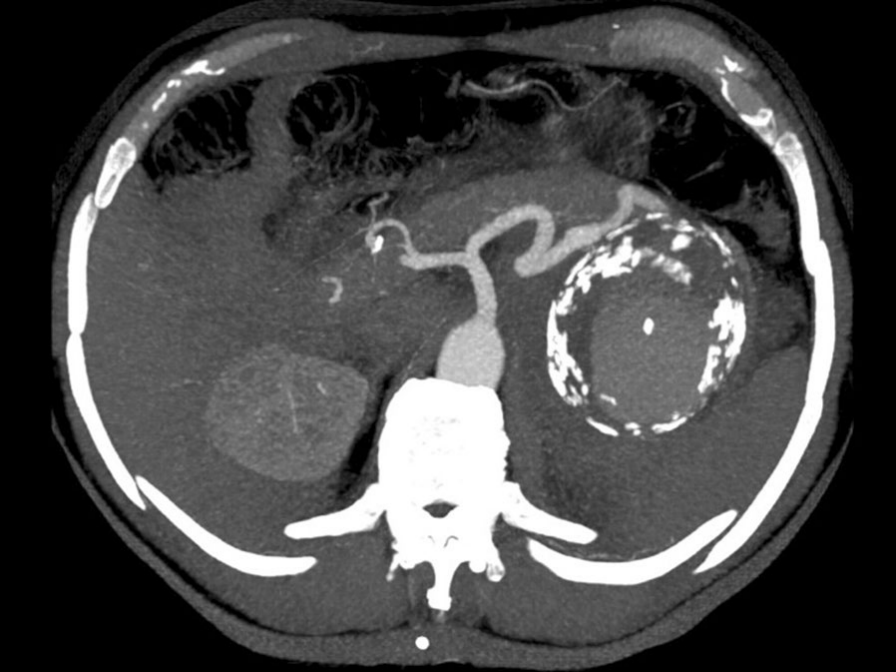
Axial arterial phase contrast-enhanced MDCT. Maximal Intensity Projection view: Giant calcified aneurysm of the splenic artery

**Fig. 4:**
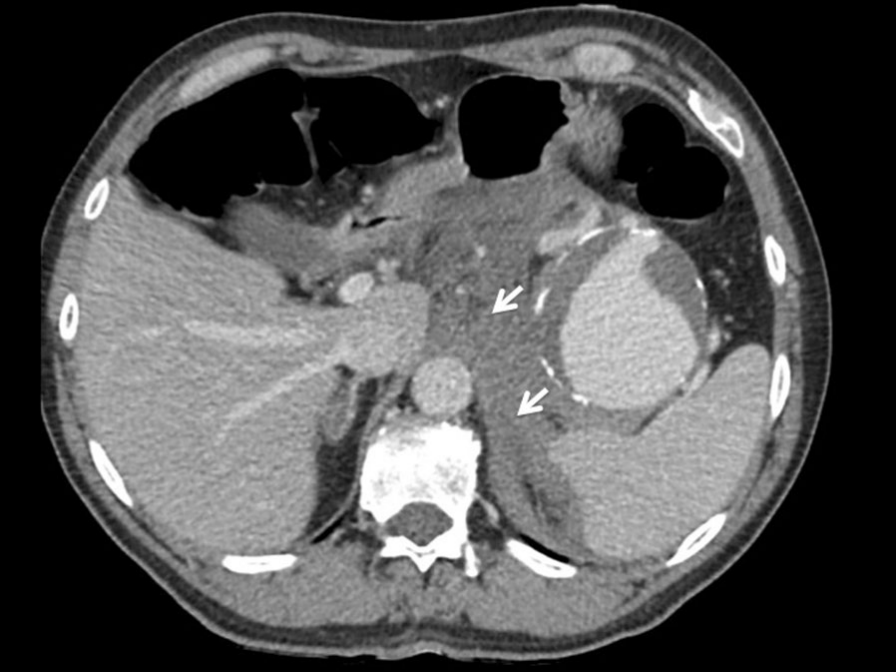
Axial portal venous phase contrast-enhanced MDCT. Hemoretroperitoneum (white arrows)

**Fig. 5:**
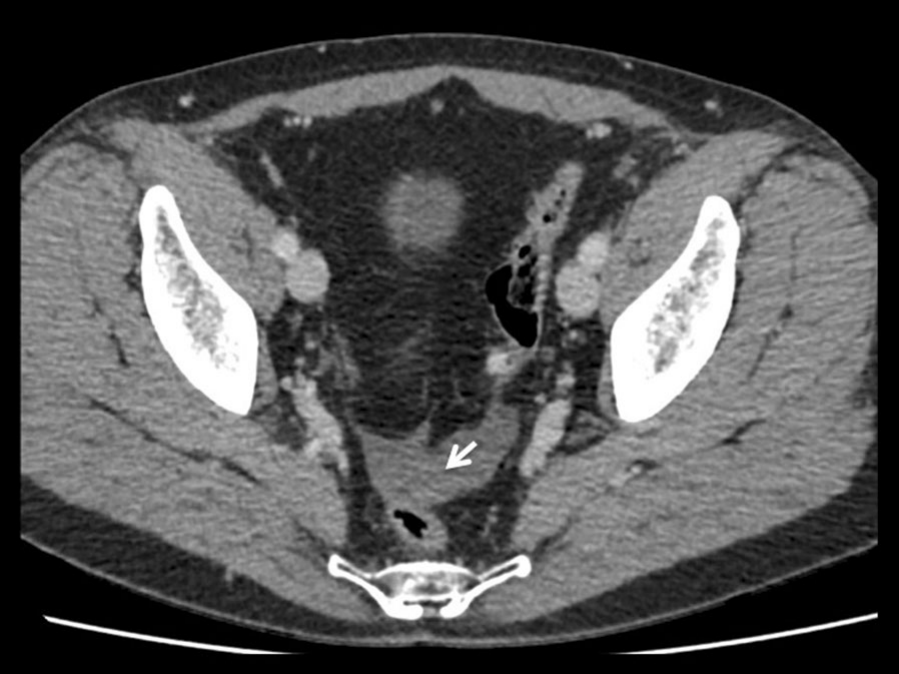
Axial portal venous phase contrast-enhanced MDCT. Pelvic hemoperitoneum (white arrow)

**Fig. 6:**
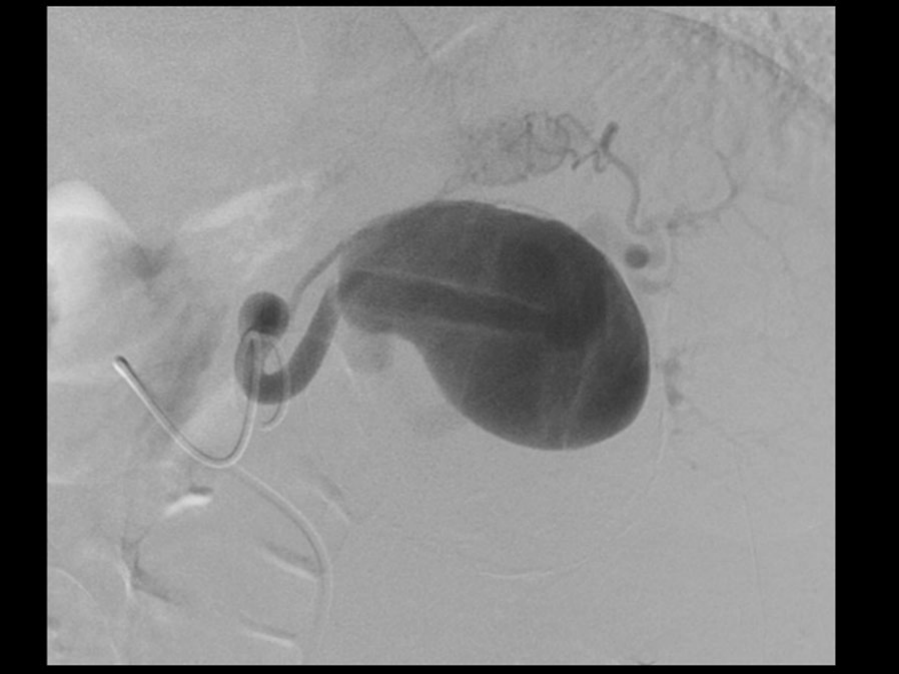
Splenic angiography. Morphological aspect of the aneurysm

Urgent computed tomography diagnosed a fissurated giant aneurysm of the splenic artery (Figs. 3, 4, 5). A selective angiography (Fig. 6) was then performed under general anesthesia; a complete exclusion of the aneurysm was obtained with glue/lipiodol embolization. The post-intervention course was uneventful and the patient was discharged home 3 days later. The patient has remained free from any complications of the embolization 6 months after the procedure.

## Discussion

We report the case of a patient presenting an acute fissuration of a previously asymptomatic giant splenic artery aneurysm detected by POCUS. Thanks to early detection, the aneurysm was embolized before full rupture and the patient was discharged home without complication. The occurrence of fissuration allowed an effective treatment before a catastrophic hemorrhagic shock. Although POCUS was used in other similar cases with SAA, the ultrasound was not conclusive and the diagnosis was made by CT-scan [2, 7]. To our knowledge, detection by POCUS of a fissuration episode has not been published. The “detection” of the SAA was done by POCUS in the case of Lo and Mok published in this journal [2], but the suspicion was of abdominal aortic aneurysm, so even if they detected it by POCUS, the correct and final diagnosis was made by CT-scan. There are many other similar cases [8] described in the literature, where POCUS showed an anechoic mass that sometimes was confused with a pancreatic cyst and others with an aortic aneurysm, and CT-scan was needed to reach the correct diagnosis [7, 9]. Rupture occurs in approximately 10% of SAA, especially when diameter exceeds 2 cm [10], with a mortality rate of 10–25% [2]. Even if incidence and rupture rates are particularly increased in the third trimester of pregnancy [10], the risk is higher from the first trimester [11]. In all patients, endovascular embolization should be considered as the first-line treatment [3, 12] rather than surgery, but strong evidence is still lacking.

Abdominal pain accounted for 8 million ED visits in USA in 2006 [1]. Epigastric pain remains a challenging situation in particular in older patients. A pragmatic pathway could include a thorough physical exam, an ECG, appropriate biological workup, and POCUS.

## Conclusion

Point-of-care ultrasound performances allow the trained Emergency Physician to rule in/out pericardial effusion [13], aortic aneurysms [14], and gallstones [15]. It is thus an invaluable tool and is recommended by the American College of Emergency Physicians [16].

## Additional files

**Additional file 1: Video 1.** POCUS scan of the left upper quadrant of a patient presenting with epigastric pain. See the vascular mass located between spleen and kidney.

**Additional file 2: Video 2.** POCUS color Doppler scan of left upper quadrant in a patient presenting an epigastric pain. See the strong Doppler signal.
